# Attention and feature binding in the temporal domain

**DOI:** 10.3758/s13423-024-02493-5

**Published:** 2024-04-08

**Authors:** Alon Zivony, Martin Eimer

**Affiliations:** 1 Department of Psychology, University of Sheffield, Sheffield, UK; 2 Department of Psychological Sciences, Birkbeck College, University of London, London, UK

**Keywords:** Attention, Binding, Attentional episodes, Temporal attention

## Abstract

Previous studies have shown that illusory conjunction can emerge for both spatially and temporally proximal objects. However, the mechanisms involved in binding in the temporal domain are not yet fully understood. In the current study, we investigated the role of attentional processes in correct and incorrect temporal binding, and specifically how feature binding is affected by the speed of attentional engagement. In two experiments, participants searched for a target in a rapid serial visual presentation stream and reported its colour and alphanumeric identity. Temporal binding errors were frequent. Critically, when participants reported the identity of a distractor instead of a target, they were also more likely to report the colour of this distractor. This association was observed both within and between individuals. These findings suggest that attentional engagement facilitates the binding of temporally co-occurring features. We discuss these results within a ‘diachronic’ framework of selective attention, and also consider other factors that contribute to temporal binding errors.

## Introduction

Perceiving objects as cohesive wholes rather than an assortment of disparate features (colour, shape) is essential to everyday functioning. Yet, how these features become bound together has not been resolved despite decades of research. Through the years, different accounts of feature binding have been put forward, most of which emphasized spatial attention as a key factor in binding (Kovacs & Harris, 2019; Treisman & Gelade, 1980; Wolfe & Cave, 1999). While the role of space in binding has been thoroughly investigated, the role of time has received much less attention. This is unfortunate since real-world visual inputs change over time and objects need to be individuated from preceding and following objects at the same location. Also, because different features are processed at different speeds (Wolfe, 2014), even spatially attended features at the same location will not necessarily be processed simultaneously, resulting in a *temporal* binding problem (Zivony & Eimer, 2022a). Similar to the phenomenon of ‘illusory conjunctions’ in the spatial domain (Treisman & Schmidt, 1982), the temporal binding problem can be demonstrated with temporal binding errors. When searching for a target among rapidly changing stimuli, features from successive objects are often incorrectly perceived as conjoined, resulting in distractor intrusion errors. For example, if participants have to detect a red digit among grey digits, they will often report seeing a temporally adjacent distractor as the red digit (e.g., Botella et al., 2001; Zivony & Eimer, 2021).

Only a few theoretical accounts of temporal binding have been put forward so far. These accounts all assume that features are sampled independently, and for binding to take place, individual features must gain access to working memory (WM). However, the role of attention for temporal binding remains disputed. According to one view (Botella et al., 2001), correct temporal binding strongly depends on an all-or-none attentional selection process. When selection occurs at the right moment, correct binding is guaranteed. In contrast, a failure of attentional selection results in both “fortunate conjunctions” (i.e., correct reports) as well as various binding errors, determined by feature salience and proximity to the target. This postulated two-stage mechanism can explain a wide range of results in distractor intrusions studies, such as differences in the pattern of intrusions when participants report colours versus identities (Botella et al., 1992). However, we have previously shown that intrusions are not associated with complete failures of attentional selection, but rather with delays in the deployment of spatial attention (Zivony & Eimer, 2021, 2023). An alternative account of binding was proposed by Vul and Rich (2010), who suggest that spatiotemporal attention plays no role in spatial or temporal feature binding. Instead, features of spatially or temporally distributed objects are sampled separately, and binding depends on which features are selected for encoding, based on probabilistic distributions. Because features are sampled independently, and their binding is not mediated by spatiotemporal attention, binding errors should show no bias towards reporting spatially or temporally co-occurring features. Reporting a feature in one dimension (identity) does not determine whether the reported feature in a different dimension (colour) belongs to the same or a different object. Vul and Rich (2010) found support for this prediction both for spatial attention with visual search tasks and for temporal attention with rapid serial visual presentation (RSVP) tasks, where participants had to report both the colour and the identity of a target (see Botella et al., 1992, for a similar task). In their RSVP task, they reported only a “negligible” (p. 1173) co-variance between the temporal positions of the reported features, suggesting that their temporal co-occurrence did not affect binding.

The conclusion that attention does not affect feature binding in space and time (Vul & Rich, 2010) is provocative, as it challenges a core assumption of current models of binding (e.g., Wolfe & Cave, 1999; Treisman, 2014). It therefore certainly deserves further critical evaluation. Here, we present two experiments that re-examined whether temporal co-occurrence affects binding, and by proxy, re-evaluate the role of attention in temporal binding. These experiments were motivated by a group of theories (Bowman & Wyble, 2007; Olivers & Meeter 2008; Shih, 2008; Sperling & Weichselgartner, 1995; Reeves & Sperling, 1986; Wyble et al., 2011) that we recently labelled as ‘diachronic’ accounts of temporal selectivity (Zivony & Eimer, 2022a). Diachronic accounts of attention – while different in many respects – all emphasize the gradual emergence of attentional selectivity across time. Specifically, they all share the assumption that perception is strongly modulated during transient periods (~150–250 ms) of attentional amplification. These “attentional episodes” (Wyble et al., 2011) are triggered once a potentially relevant event is detected, and then indiscriminately modulates the perception of all the features that appear in the same location, thereby increasing the likelihood that they will be encoded. Because the processing of multiple objects is likely to be enhanced during the attentional episode, a distractor feature may be encoded instead of the corresponding target feature, resulting in an intrusion error. However, and in direct contrast to the proposal by Vul and Rich (2010), the distinct time course of amplification during an attentional episode should result in a dependency in the reports of features from the same object. Because amplification is indiscriminate, when two features appear at the same time, their processing should benefit to a similar extent from the amount of amplification received at that point in time. Moreover, since the onset of the attentional episode is not fixed, but rather varies from trial to trial (Zivony & Eimer, 2021), which features receive maximal attentional enhancement changes from trial to trial. Therefore, participants should be biased towards reporting the target’s feature (or the features of the object preceding the target) when the attentional episode is triggered early, and they should be biased towards reporting the features of the following object(s) when the attentional episode is triggered late. Thus, diachronic accounts predict that there should be a bias towards encoding and reporting features from the same object in RSVP tasks.

Vul and Rich (2010) may have failed to observe such a dependency because several aspects of their experimental design and analysis could have reduced the likelihood to observe such an effect. In the current Experiment 1, we employed their general design, but changed some key features that may have obscured evidence for object-based feature binding (see below). Importantly, Vul and Rich examined co-variation in position of reported features across all trials and all participants (i.e., each trial was a datapoint). This analysis ignores individual differences in binding that may be linked to known individual differences in temporal selectivity (Martens et al., 2006). It is possible that some participants showed object-based feature binding, but this may have been obscured by the data from other participants who did not. Therefore, the current study included additional analyses that take such individual differences into account. We predicted that with this analysis a robust dependency will emerge between reports of co-occurring features.

## Experiment 1

### Method

**Fig. 1:**
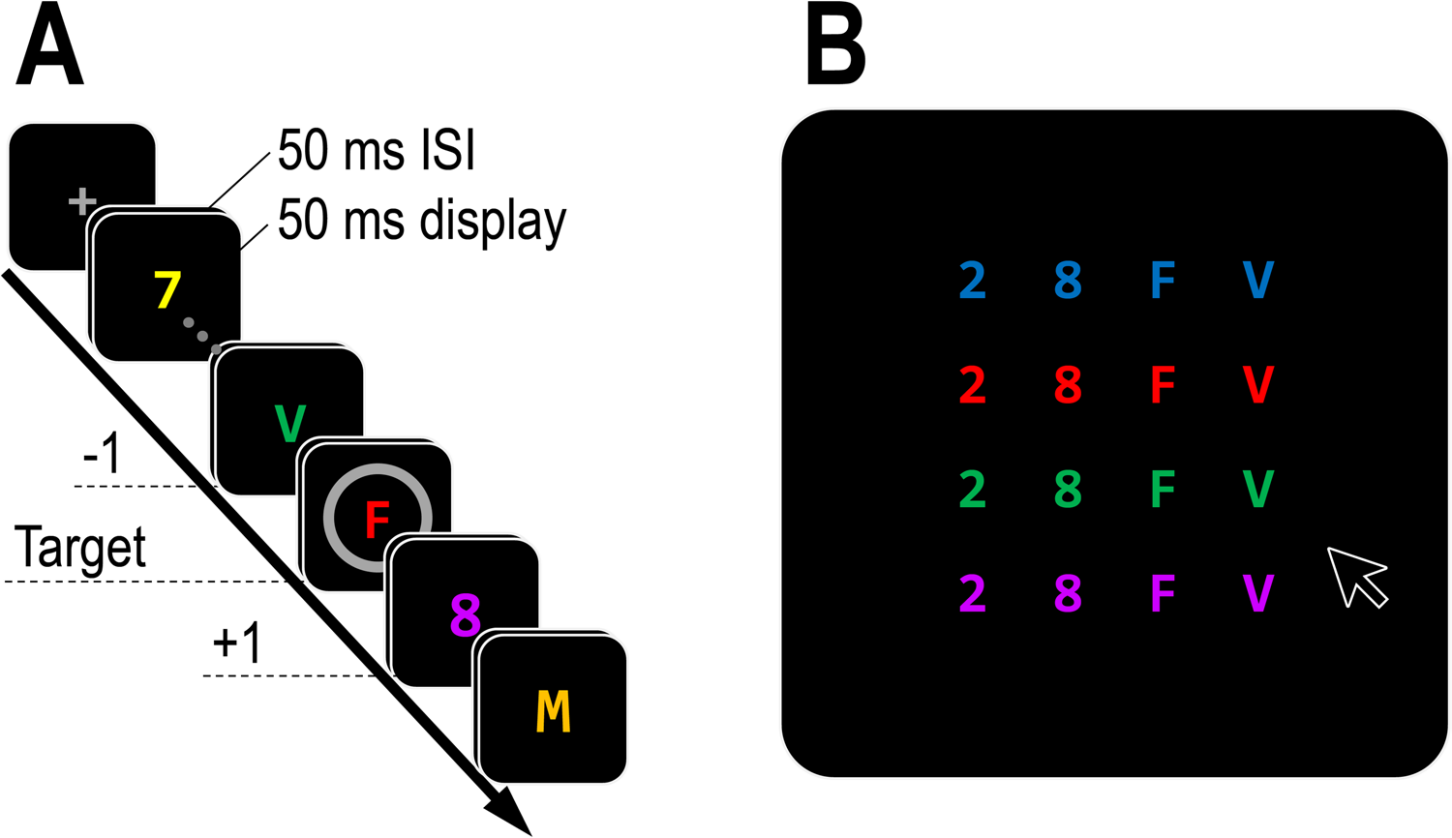
Illustration of the experimental paradigm used in Experiment 1. Participants had to find a target character inside a circle (**A**), embedded in a rapid serial visual presentation (RSVP) stream of grey digits and letters, and report its colour and identity (**B**). The response screen always included the identities and colours of the target, the pre-target (-1) distractor, post-target (+1) distractor, as well as one identity (e.g., “2”) and one colour (e.g., blue) that did not appear in the RSVP

In their experiment, Vul and Rich (2010) asked participants to report a target’s identity and colour in two sequential responses. This may have weakened evidence of the importance of co-occurrence because participants could not match the stimuli in the response screen based on familiarity or a full match with a memorized object. Furthermore, their response screens included the target and two preceding and following distractors, but no unrelated items, so that guess rates could not be quantified. In Experiment 1, all target/distractor colour and identity combinations were presented in the same response screen, and these screens also included a “foil” object that was not present in the RSVP stream (Fig. 1B). Thus, response choices could be based on full matches and/or familiarity, and the probability of guesses could be assessed.

#### Ethics

All methods used in this and the next experiment were approved by the institution’s departmental ethical guidelines committee at Birkbeck, University of London.

#### Sample size selection

We based our sample size on Vul and Rich (2010), even though we could not use their report to conduct a formal power analysis. They reported that with 14 participants and 100 trials per condition (i.e., 1,400 observations), they observed a negligibly small but significant correlation between reports of identity and of colour in their RSVP task (100-ms condition). To increase power, we increased the number of participants to 20 and increased the number of observations by 2.4 (to a total of 4,800 observations). This ensured that the current study has enough power to detect even smaller effects than the ones observed in Vul and Rich.

#### Participants

After the exclusion of a single participant from the dataset (see below), the sample included 20 (16 women) volunteers (*M*_*age*_ = 27.7, *SD* = 7.1 years) who participated for £5 or course credits. All reported normal or corrected-to-normal visual acuity and normal colour vision. Participants were given the option to report gender identities other than woman or man. In this experiment and all subsequent experiments, these options were not selected. No other demographic information was collected.

#### Apparatus

The experiment was conducted using participants’ individual computers, who accessed and downloaded the experiment via E-Prime Go cloud service. Subjects were asked to sit approximately 60 cm from the screen (approximately an arm’s length), in a quiet and distraction-free environment, and complete the task in one sitting within 35 min. Manual responses were given via standard keyboard and mouse.

#### Stimuli, procedure and design

All stimuli sizes were calculated in visual angles based on the participants’ self-reported monitor size (monitor sizes ranged from 12 in. to 17 in.) and an assumed distance of 60 cm from the screen. If participants did not know their monitor size, they were directed to a website that calculates it for them (www.piliapp.com/actual-size/credit-card/).

Participants had to report as accurately as possible the identity of an alphanumeric character that appeared inside a (0.8° radius) circle cue (the selection feature). These targets were presented unpredictably in an RSVP stream that appeared in the centre of the screen. Manual responses were executed without time pressure at the end of each trial. The sequence of events is illustrated in Fig. 1A. Each trial began with the presentation of a fixation display (a grey 0.2° × 0.2° “+” sign at the centre of the screen). Then, after 500 ms, the RSVP stream appeared.

The target digit appeared with equal probability and unpredictably in the sixth, eighth, tenth or 12th frame within the RSVP stream, and was followed by two additional distractors. Therefore, the length of the RSVP was between eight and 14 frames. Alphanumeric characters were all 1.3° in height. The selection cue had a radius of 0.8° and line width of 4 pixels. All characters in the RSVP streams were grey and were randomly selected without replacement from a 24-letter set (all English alphabet letters, excluding I and O) and a set of eight digits (2–9), with the restriction that letters and digits appeared equally often in the RSVP. All characters were drawn in ‘Consolas’ font. The letters and digits were drawn in one of six possible colours: green (RGB values: 0,90,0), blue (50,100,255), orange (255,175,200), yellow (255,255,0), magenta (160,75,160) and red (255,0,0). On every trial, one colour did not appear in the RSVP stream. The colour on each frame was randomly drawn from the remaining five colours with the following restrictions: colours could not repeat until all five colours appeared and could not appear on two frames in a row. Finally, the colours of the distractor that preceded and the two distractors that followed the target (-1, +1, and +2 positions) were always different from one another and different from the target’s colour.

Each frame appeared for 50 ms, followed by an interstimulus interval (ISI) of 50 ms. E-prime Go can collect data about exact presentation times, which varied across different computers. Participants were excluded if their monitor’s refresh rate could not produce these stimulus durations or ISI durations (e.g., if their monitor refresh rate was 50 Hz, they were not included in the sample; see Zivony & Eimer, 2022b, for a similar procedure). After the exclusion of a single participant, each frame appeared on average for 49.80 ms (*SD* = 0.45 ms), followed by an ISI of 49.86 ms (*SD* = 0.76 ms).

The response screen (see Fig. 1B) included a 4 × 4 grid. Columns were based on different colours. They included, in random order, the colour of the target, the colour of the pre-target (-1) distractor, the colour of the post-target (+1) distractor, and the colour that did not appear in the RSVP on that trial. Rows were based on different identities. They included the identities of the target, pre-target (-1), post-target (+1), and another digit or letter that did not appear in the RSVP stream. From left to right, digits were presented first (sorted based on size) and letters later (sorted based on alphabet order). Thus, the locations on the grid were uncorrelated with the order in which the colours or identities appeared in the stream. The centre-to-centre distance between characters was 3.0° both horizontally and vertically. Participants used the mouse to select one of the characters, by pressing on an area within an invisible 0.8° × 1.0° rectangle around a character. Once pressed, a (0.8° radius) circle appeared for 200 ms around the selected character to provide participants with visual feedback that their response was registered. Following feedback, a blank screen appeared for 800 ms before a new trial started. The experiment included ten practice trials followed by 240 experimental trials, divided into 60-trial blocks.

#### Analysis

For any given trial, we coded which features participants reported based on their temporal position relative to the target (a position index). That is, reporting a target feature yields a position index value of 0 and reporting the distractor immediately following or preceding the target yields a value of +1 and -1, respectively. Our first analysis was meant to replicate Vul and Rich’s results. Therefore, we conducted a Spearman Rho’s correlation analysis on the position of the reported colour (-1, 0, or +1) and the position of the reported identity (-1, 0, or +1), taking into account all trials except for trials with foil reports. This analysis does not take into account individual differences, which might be an important source of statistical error. Therefore, our second and third analyses used a within-subject design.

Our second analysis compared the likelihood of making different types of errors (Dowd & Golomb, 2019).^1^ If co-occurring features are processed independently, reporting of one of the distractor’s features should not be correlated with reporting its other features. For example, report of the -1 identity should be equally likely to be accompanied with report of the -1 colour (correlated intrusion) or of the +1 colour (uncorrelated intrusion). In contrast, if there is a dependency between co-occurring features, correlated intrusions should be substantially more frequent than uncorrelated intrusions.

A drawback of this analysis is that it excludes a large proportion of trials (i.e., trials where target features were reported). Therefore, we also examined the dependency between identity reports and colour reports with two additional methods. First, we quantified the average reported feature for each participant as a function of the feature’s position relative to the target – an average position index (API; see also Botella et al., 2001). For each participant, we calculated the average position of colour reported (colour API) as a function of the reported identity (-1, 0, or +1). Then, colour APIs were compared using a repeated-measures ANOVA with the reported identity as the independent variable.^2^ Following our previous findings showing that report of distractor identities depends on the onset of the attentional episode (Zivony & Eimer, 2021), we predicted that colour APIs would change as a factor of the reported identity: they should be higher on trials where participants report the identity of the +1 distractor than the target or the -1 distractor. In contrast, a lack of dependency between the two measures should result in similar colour API regardless of the reported identity. A significant main effect was followed by Bonferroni-corrected comparisons that examined the differences in colour API between each pair of reported identities (pre-target vs. target, target vs. post-target).

Finally, another way to examine the dependency between temporally co-occurring features is to use an individual differences approach. If the timing of attentional episodes plays a role in binding and varies reliably between different individuals, then some individuals should have a high-identity API and high-colour API, whereas others should have relatively low API on both measures. In contrast, if the timing of attentional episodes does not play a role in binding and/or does not reliably vary between participants, then participants with a high-identity API should be equally likely to show a low-colour API. To examine this, we calculated the average colour API and identity API for each participant and then calculated the Pearson correlation between the two measures.

### Results

**Fig. 2:**
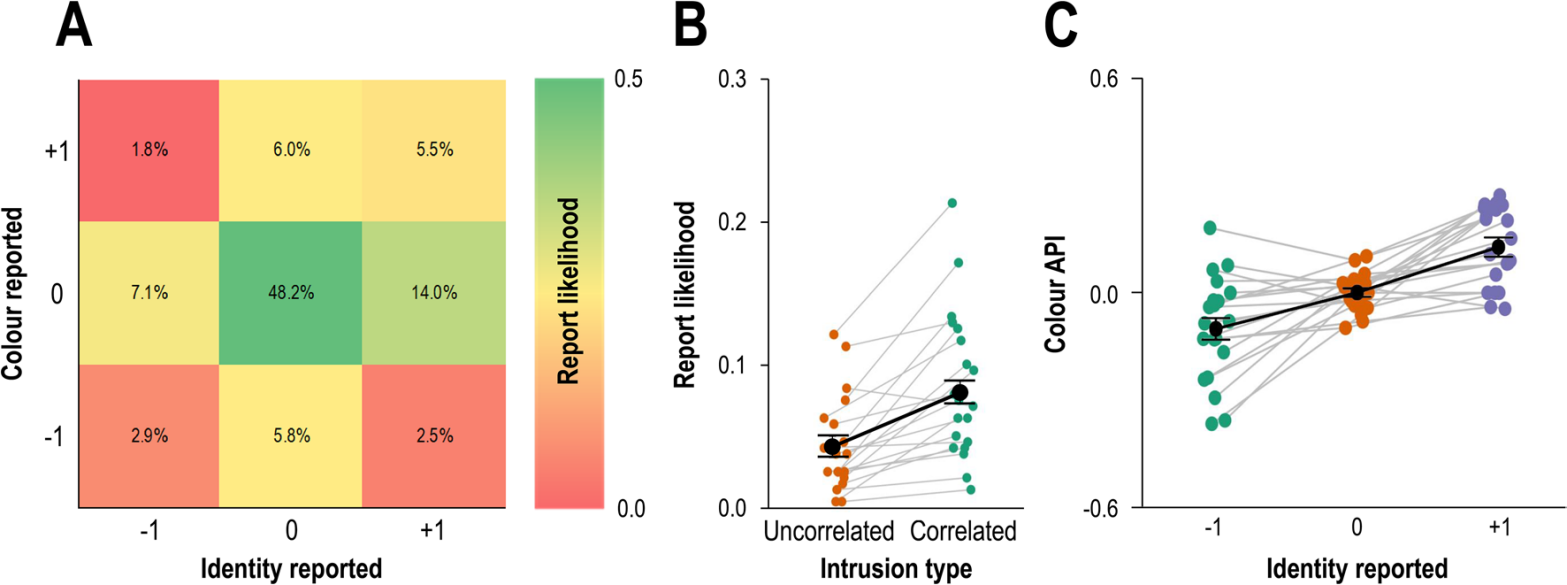
Results from Experiment 1. (**A**) Frequency table denoting the report frequency of each of the possible conjunctions (reported colour and reported identity), except for foil reports. (**B**) Report likelihood of reporting a combination of distractor features from two different distractors (uncorrelated intrusion) versus the likelihood of reporting two distractor features from the same distractor (correlated intrusions). (**C**) Average reported position in the colour dimension (average position index, API) as a function of the reported identity. In panels B and C, the black line reflects the average across all participants (error bars reflect one standard error) and grey lines reflect individual participants

Participants mostly reported the target accurately (48.2%; Fig. 2A centre) or else reported a combination of one of the target’s features and one of the distractor’s features (32.9%; Fig. 2A, sum of datapoints perpendicular to centre). The likelihood to report the foil was very low, *M* = 2.4% and *M* = 4.0%, for identity and colour reports, respectively. On average, participants were less accurate in identifying the target’s identity than the target’s colour, *M* = 61.8% versus *M* = 70.5%, *t*(19) = 3.48, *p* = .002, *d*_*z*_ = 1.23. The full distribution of reported features (except for foil reports) is presented in Fig. 1A. As predicted, the first analysis yielded a small yet significant positive correlation between the position of the reported colour and identity, *ρ*(4690) = .13, *p* < .001 (Fig. 2A).

#### Within-participants dependency

Participants were almost twice more likely to make correlated intrusion errors (reports of both features from the same distractor; Fig. 2A, bottom-left and top-right corners) than uncorrelated intrusion errors (reports of features from different distractors; Fig. 2A, bottom-right and top-left corners), *M* = 8.4% versus *M* = 4.3%. This observation was confirmed by a direct comparison of these two types of errors, *t*(19) = 5.24, *p* < .001, *d*_*z*_ = 1.17 (Fig. 2B). Moreover, colour API gradually rose as a function of the position of the reported identity (Fig. 2C): colour API was lowest when participants reported the pre-target identity (*M* = -0.10), higher for target reports (*M* = -0.001), and highest for post-target reports (*M* = 0.13). The third analysis confirmed this observation, *F*(2,40) = 24.86, *p* < .001, ${\eta }_{p}^{2}$= .55. The follow-up analysis indicated that differences between colour API on each pair of reported identities was significant, *t*(19) = 2.96, *p* = .008, *d*_*z*_ = 0.90, and *t*(19) = 4.06, *p* < .001, *d*_*z*_ = 1.24.

#### Between-participants dependency

**Fig. 3:**
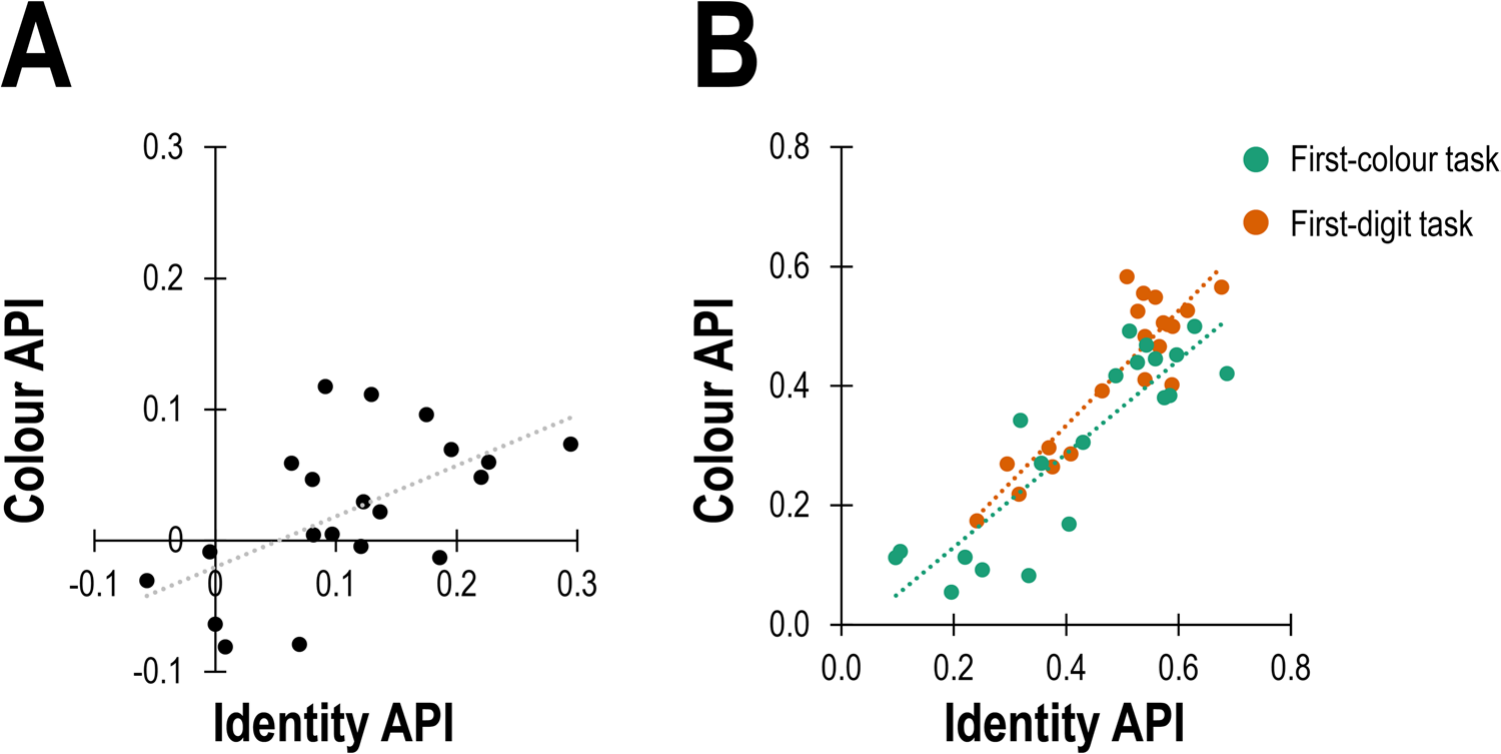
Scatterplot of average reported colour position (colour average position index, API) and average identity position (identity API) in Experiment 1 (**A**) and Experiment 2 (**B**). Data in Experiment 2 is separated as function of the task (first-colour vs. first-digit). Each dot reflects one participant. The dotted line reflects the linear regression line fitted based on the data

The fourth analysis revealed a significant positive correlation between average colour APIs and average identity APIs, *r*(18) = .58, *p* = .007 (Fig. 3A).

### Discussion

The results of Experiment 1 demonstrated that temporally co-occurring features are more likely to be encoded together, as reports of an object’s identity were associated with a higher likelihood of reporting the same object’s colour. Importantly, participants who had a high API on one feature also had a high API on the other. Once these individual differences were accounted for, the effect sizes demonstrating co-dependence of feature reports were far from negligible. At the same time, binding errors still occurred on a substantial number of trials. We return to this point in the *General discussion*. Another finding was that colour reports were more accurate than identity reports. This is compatible with the notion that colour is processed faster than semantic identity (Treisman, 2014; Wolfe, 2014), and that this difference affects feature binding.

However, two issues potentially limit these conclusions. First, stimulus set size differed between dimensions, as there were 32 possible letters and only six possible colours, which may have contributed to the observed asymmetry between these dimensions. More importantly, the shape selection feature in Experiment 1 was not part of the reported object itself. Thus, the observed dependency between co-occurring features may have resulted from the additional temporal demands of shifting attention from the selection cue to the object inside the stream. Experiment 2 was designed to address these issues.

## Experiment 2

### Method

In Experiment 2, target set size was limited to four possible colours and four possible identities. Importantly, instead of searching for a shape cue, participants were instructed to report either the first coloured item after a series of grey distractors or the first digit following a series of distractor letters. Thus, the selection feature was now always a part of the reported object. As before, the question was whether reliable evidence for the preferential binding of temporally co-occurring features would be obtained under these circumstances.

#### Sample size selection

We conducted a power analysis to calculate the required sample to replicate the main result (Fig. 2B) in Experiment 1 with 80% power. To do this, we entered the effect size of the one-way ANOVA (${\eta }_{p}^{2}$=.55) to G*Power (Faul et al., 2013). This analysis revealed that a sample of a mere seven participants would suffice to replicate this effect. To allow for a better comparison between the two experiments, we once again recruited 20 participants.

#### Apparatus

Unlike Experiment 1, this experiment was conducted in the lab and not in participants’ homes. Stimuli were presented on a 24-in. BenQ LED monitor (120 Hz; 1,920 × 1,080 screen resolution) attached to a SilverStone PC, with participant viewing distance at approximately 80 cm. Manual responses were registered via a standard mouse.

#### Stimuli, procedure and design

The stimuli, procedure and design were the same as Experiment 1 except for the following changes. For every participant, the set of potential target stimuli was comprised of a combination of four possible digits in four possible colours. The set of four digits was selected at random at the beginning of the experiment from the set used in Experiment 1 and remained the same throughout the experiment. Participants were notified that the target will always be one of these digits. The four colours were orange (CIE colour coordinates: .476/.462), green (.247/.402), blue (.199/.253) and magenta (.333/.165), and were matched in luminance (38.8–40.3 cd/m^2^).

**Fig. 4:**
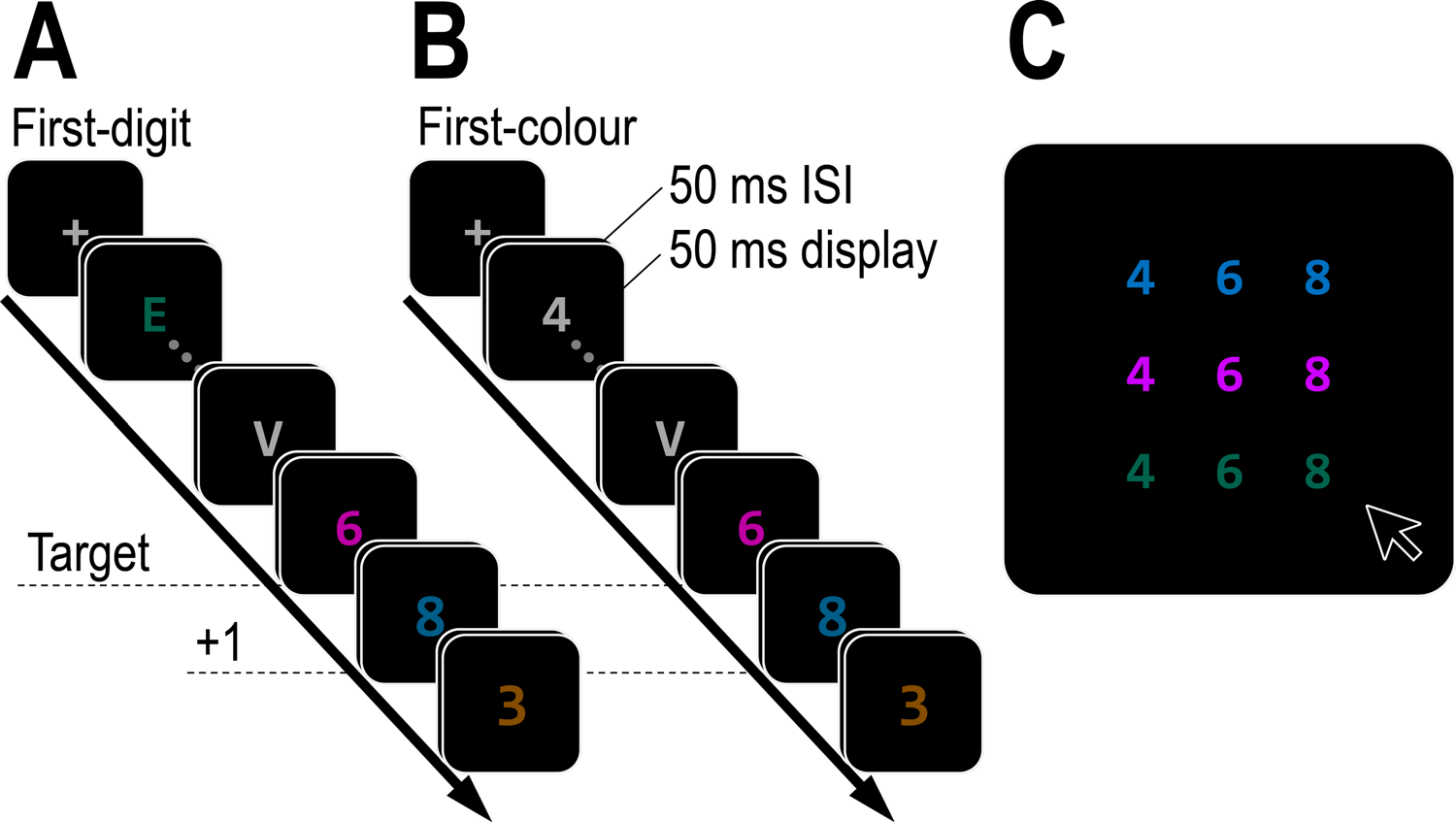
Illustration of the experimental paradigm used in Experiment 2. Participants had to find the first digit in a stream of differently coloured letters (**A**) or the first coloured item (**B**) among a stream of grey coloured digits and letters, and report its colour and identity. The response screen (**C**) always included the identities and colours of the target, the post-target (+1) distractor, as well as a foil colour (e.g., green) and a foil digit (e.g., “4”) that did not appear near the target

The experiment included two tasks. In the *first-digit* task (Fig. 4A), participants had to detect the first digit in the RSVP, whereas in the *first-colour* task (Fig. 4B), they had to detect the first coloured item in the RSVP. The two tasks had the following in common: the target was always a coloured digit, and participants had to report both its identity and colour. The target was always followed by two frames of differently coloured digits. The response screen always included nine options, sorted in a 3 × 3 array (Fig. 4C). These included the target digit, the post-target (+1) digit, and a foil digit that did not appear near the target (i.e., not in the -1 or +2 positions), and these items were presented in the target’s colour, the post-target colour, and a foil colour.

The main difference between the tasks was the RSVP frames that preceded the target. In the first-digit task, the target was preceded by differently coloured letters. The letter identities were selected randomly without replacement from the set used in Experiment 1. Half of these letters were grey, whereas the others were coloured randomly in one of four possible colours. The sole restrictions were that the same colour could not repeat twice on two consecutive frames, and that the distractor immediately preceding the target could be either grey or match the +2 distractor (i.e., it did not share the target’s colour, the post-target colour, or the foil’s colour).

In contrast, in the first-colour task, the target was preceded by grey digits and letters. The letter identities (50% of distractors) were selected randomly without replacement, whereas the digit identities were randomly selected with replacement from the set of four possible digits. The sole restrictions were that the same colour could not repeat twice on two consecutive frames, and that the distractor immediately preceding the target could only be a letter or match the +2 distractor (i.e., it did not share the target’s identity, the post-target identity, or the foil’s identity). Taken together, the difference between the two tasks was in what information participants could use to search for the target. Even though the target was always a coloured digit, participants could only utilize the target’s alphanumeric category in the first-digit task (since many distractors were coloured) and could only utilize the target’s colour in the first-colour task (since many distractors were digits).

Participants completed eight blocks of 60 trials (i.e., a total of 480 trials), divided into two halves based on the task. The order in which the tasks was presented was counterbalanced between subjects. Before the experiment began participants received instructions and were given ten practice trials. At the halfway point, they received instructions regarding the new task and were given five practice trials.

#### Analysis

We once again examined the dependency between identity reports and colour reports, and conducted this analysis separately for both tasks. To do so, we excluded trials where participants selected the foil in either reporting dimensions. Like Experiment 1, we first conducted a Spearman Rho’s correlation analysis on the position of the reported colour (0 or +1) and the position of the reported identity (0 or +1). In this experiment, the -1 distractor was not reportable, and therefore we could not compare between correlated and uncorrelated intrusions. For the second analysis, we once again examined the average colour API as a function of identity report (target vs. post-target). We entered these APIs as a dependent variable in a two-way repeated-measures ANOVA with task (first-digit vs. first-colour) and reported identity (target vs. post-target, i.e., 0 vs. +1). Finally, like Experiment 1, we examined the correlation between average identity API and average colour API using a Pearson correlation.

### Results

**Fig. 5:**
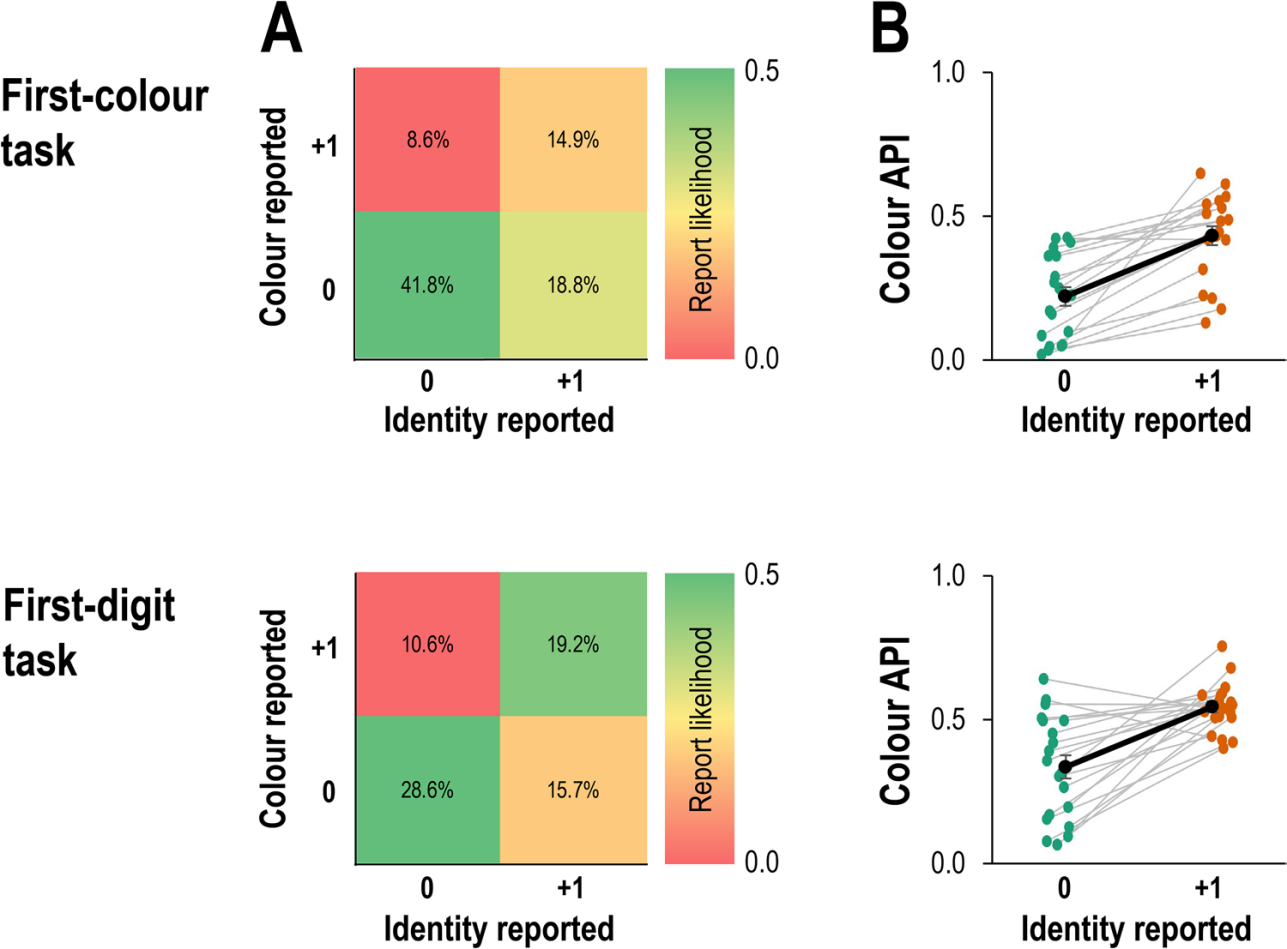
Results from Experiment 2 as function of the task (first-colour vs. first-digit, reported in the upper and lower row respectively). (**A**) Frequency tables denoting the report frequency of each of the possible conjunctions (reported colour and reported identity), excluding foil reports. (**B**) Average reported position in the colour dimension (average position index, API) as function of the reported identity. The black line reflects the average across all participants (error bars reflect one standard error) and grey lines reflect individual participants

Performance was generally poorer in the first-digit task than the first-colour task. This can be seen from the accuracy level in reports of both identity (*M* = 39.2% vs. *M* = 50.4%; Fig. 5A, sum of left columns) and colour (*M* = 44.3% vs. *M* = 60.6%; Fig. 5A, sum of bottom rows), both *p*s < .01. Similarly, foil identities and foil colours were more likely to be reported in the first-digit task than in the first-colour task (*M* = 13.9% vs. *M* = 10.4% and *M* = 15.5% vs. *M* = 7.4%, respectively), both *p*s < .05. In both tasks, correct reports were most common, followed by incorrect bindings of colour and identity, and reports of the post-target distractor (Fig. 5A). The first analysis revealed a significant correlation between the positions of reported colours and identities for both the first-digit and the first-colour tasks, *ρ*(3677) = .29, *p* < .001 and *ρ*(4164) = .30, *p* < .001, respectively.

#### Within-participants dependency

The second analysis revealed that APIs were higher in the first-digit task than in the first-colour task (*M* = 0.44 vs. *M* = 0.32), *F*(1,19) = 27.39, *p* < .001, ${\eta }_{p}^{2}$= .59. Importantly, colour APIs were also higher on trials where participants reported the +1 distractor identity (*M* = 0.55 vs. *M* = 0.34), *F*(1,19)=37.66, *p*<.001, ${\eta }_{p}^{2}$=.67 (Fig. 5B). The interaction between the two factors was not significant, *F*<1.

#### Between-participants dependency

The correlation between identity API and colour API was significant for both the first-colour and the first-digit tasks, *r*(18) = .88, *p* < .001 and *r*(18) = .89, *p* < .001 (Fig. 3B).

### Discussion

The results of Experiment 2 closely replicated those of Experiment 1,^3^ even though selection cues and response feature belonged to the same object. This demonstrates that the dependency between co-occurring features observed in Experiment 1 was not a result of the need to realign the focus of attention between two different objects. Interestingly, the nature of the selection cue did not systematically affect reports. For example, when participants were searching for the first digit, they still reported the post-target digit identity on 41.7% of the trials, which suggests that the selection feature does not automatically receive priority in encoding. This is in line with our own recent diachronic account (Zivony & Eimer, 2022a), which does not assume that selection features have a special status in encoding. Furthermore, the findings from Experiment 1 were replicated even though target set sizes were equalized for colour and identity. Thus, the asymmetry between identity and colour reports likely reflect differences in processing speed (Wolfe, 2014).

## General discussion

Binding is a fundamental process that allows objects to be perceived as coherent events, rather than disparate features. Although it is generally believed that attention plays a crucial role in binding (Kovacs & Harris, 2019; Treisman & Schmidt, 1982), findings by Vul and Rich (2010) have suggested that this is not the case, for binding both in space and in time. In the two experiments presented here, we re-assessed their provocative conclusion for the case of feature binding in the temporal domain. We used procedures similar to Vul and Rich (2010), with some modifications to the methods and analysis.

**Fig. 6:**
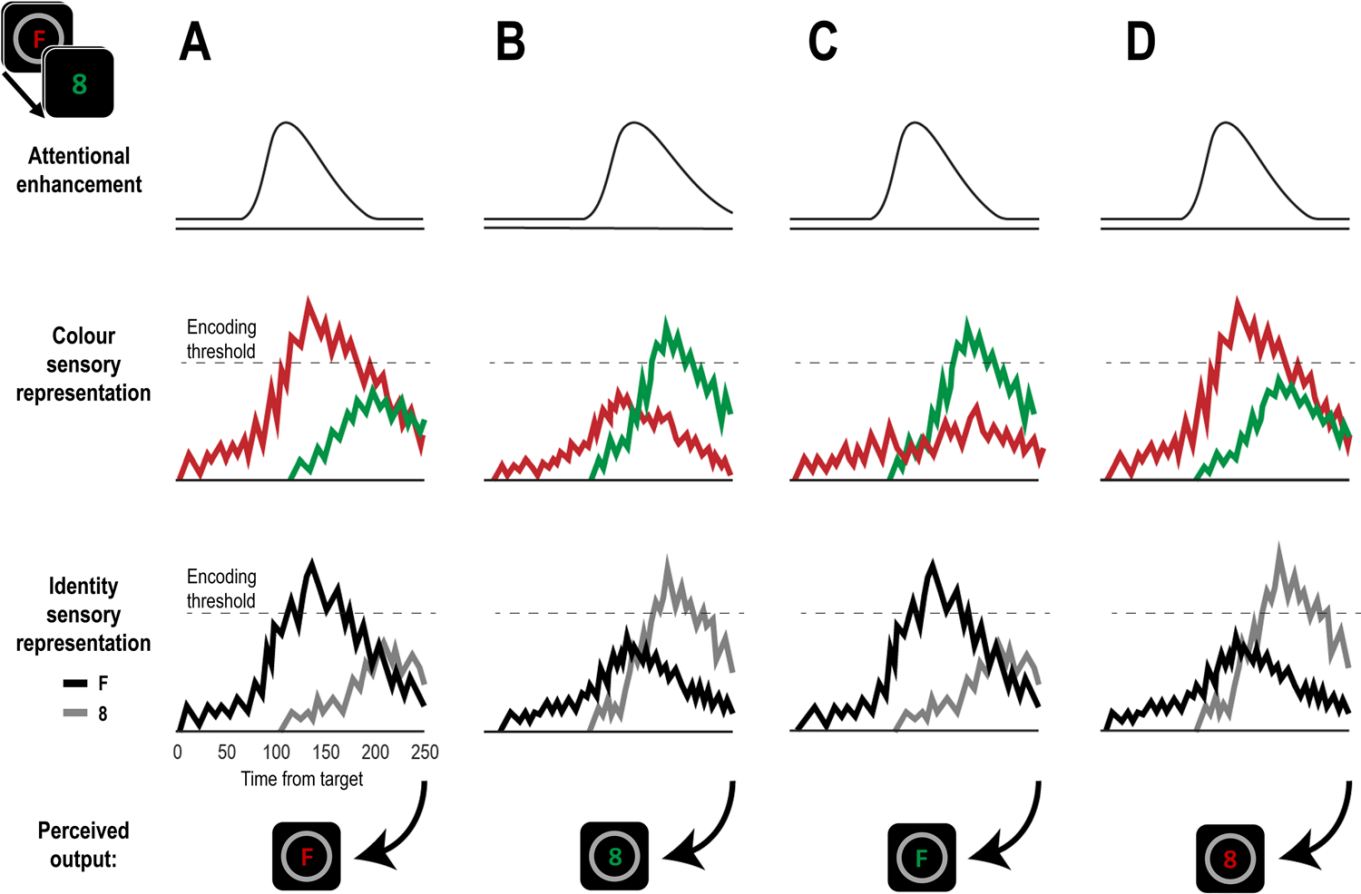
Illustration of factors that determine temporal feature binding in the diachronic account on hypothetical trials. In this example, the selection feature is a circle, the target is a red “F”, and the post-target distractor is a green “8”. The x-axis in each panel represents time in milliseconds from the moment signals from the target reach the visual cortex. Evidence about each feature (colour and identity) is accumulated separately and continuously modulated by spatially-specific attentional enhancement. In addition, sensory representations mutually inhibit one another. Once the target is detected, it triggers an attentional episode. When this attentional episode is triggered early (**A**), it is more likely that both the target’s features will be sufficiently strong to cross the encoding threshold and be encoded. When the attentional episode is substantially delayed (**B**), there is a higher likelihood that both of the post-target’s features will be encoded instead. However, high perceptual noise in one feature (**C**) or different rates of evidence accumulation due to different processing speed (**D**) can result in temporal misbinding where the perceived object is comprised of two features from two different objects

Participants had to detect a target and report both its colour and identity. Results consistently showed that the co-occurrence of features increased the probability of their binding into a single object. When participants erroneously reported the identity of a distractor, they were also more likely to report the same distractor’s colour. This association was substantially strengthened when individual differences and trial-by-trial variability were accounted for. Together with our previous observation that intrusion errors are associated with a delayed onset of the attentional episode (Zivony & Eimer, 2021), these findings show that the timing of attentional engagement affects all features presented in that point in space and time. When engagement is fast, there is a high likelihood that the processing of all target features will be sufficiently enhanced to be encoded together (Fig. 6A). When engagement is slow, features from the distractor object following the target will be encoded instead (Fig. 6B). This conclusion is further supported by the observation that intrusions were strongly affected by the type of selection cue being employed. In Experiment 2, intrusions were lower in the first-colour task than in the first-digit task, plausibly because colours were detected more quickly, making the colour-defined target less vulnerable to masking. Likewise, the probability of post-target intrusions in Experiment 1 was lower than in Experiment 2, plausibly because, on average, the onset selection cue was detected more rapidly. Interestingly, dependency between co-occurring features was also lower in Experiment 1. It is possible that when attentional cues are highly salient, attentional episodes are not just triggered faster but are also less variable in their timing, thereby limiting the potential for detecting shared variance between features. Given the other differences between the two experiments (see footnote 3), this possibility remains speculative, but points to potential new avenues of research about the relationship between the timing of attention and feature binding. In any case, in contrast to the probabilistic independent feature sampling account proposed by Vul and Rich (2010), we conclude that attentional mechanisms, and in particular the temporal dynamics of spatial attention, have a direct impact on the process of combining features from different dimension. They do so by making it more likely that co-occurring features will be perceived to belong to the same object.

While co-occurrence plays an important role in temporal binding, it is not always sufficient to guarantee correct binding. In the current study, participants often reported the target colour or identity alongside the identity or colour of a temporally proximal distractor. This shows that while attentional factors facilitate the dependence between co-occurring features, other factors can result in the independent encoding of features from different objects. In our diachronic account (Zivony & Eimer, 2022a), perception is described as a process of evidence accumulation that is modulated by attention, particularly during attentional episodes. As a result, some features (usually those that benefitted from attentional modulation) reach the threshold required for encoding. However, evidence accumulation is also affected by attention-unrelated factors. One of these relates to feature-specific variations in perceptual noise (Ashby & Lee, 1993), which can affect evidence accumulation, allowing for features from other objects to “win the race” for encoding (Fig. 6C). This view is compatible with the finding that averaging responses across individual trials resulted in much larger effect sizes, as averaging reduces the effect of perceptual noise and better reflects the central tendency of the real effect for each participant.

Binding errors can also occur when different features are processed at different speeds. In this case, the feature that is processed more slowly is more vulnerable to intrusions, resulting in more errors on this dimension (Fig. 6D). In the current experiments, this was illustrated by the asymmetry between colour and identity reports. In both experiments, target colour/distractor identity reports were more frequent than target identity/distractor colour reports (Experiment 1: *M* = 21.1% vs. *M* = 11.8%; Experiment 2 first-colour task: *M* = 18.8% vs. *M* = 8.6%; Experiment 2 first-digit task: *M* = 15.7% vs. *M* = 10.6%, all *p*s < .001). Finally, while fast attentional engagement is likely to result in correct reports and delayed engagement in reports of the post-target distractor object, intermediate speeds of attentional engagement could result in an encoding of multiple features from different objects (Vul et al., 2009), at the expense of precise temporal information (Akyürek & Wolff, 2016; Akyürek et al., 2012). In such cases, perceptual decisions may indeed follow a probabilistic distribution, as suggested by Vul and Rich (2010), and these should be associated with substantially reduced confidence (Recht et al., 2019) relative to trials where only a single object is encoded.

In contrast to most previous work on the role of attention in object binding, which were focused on spatial factors (e.g., Treisman & Schmidt, 1982), the current study investigated temporal binding. It is important to note that attention is likely to operate differently in these two dimensions, due to the way that space and time are processed in the visual system. Because the visual cortex is retinotopically organised, interference from equidistant distractors will be similar regardless of their spatial position relative to the target (Klein et al., 2023). In contrast, time perception is an inferred property based on processing of multiple events (e.g., Block & Gruber, 2014), and the effects of temporal proximity on binding are asymmetrical, with generally larger interference by stimuli that follow the target relative to preceding objects (e.g., Botella et al., 2001; Klein et al., 2023). This asymmetry is easily explained by the diachronic account, as attentional episodes are only triggered once sufficient evidence for the presence of a potential target has accumulated. By that time, representations of previous stimuli may already have faded or been overridden by the target object. This implies that manipulations that delay attentional engagement should result in more post-target distractor intrusions, while manipulations that speed up engagement should result in better accuracy but no increase of pre-target intrusions (as has indeed been found; Zivony & Eimer, 2023).

Given these differences, prior observations regarding binding in space do not necessarily apply to the role of attention in temporal binding. This underlines the need for additional research in this field, which needs to address several unanswered questions. For example, the robust individual differences observed here go beyond previous studies (e.g., Martens et al., 2006) to reveal undocumented variability in the speed of attentional engagement. This variability may produce important differences in real-world behaviour that depends on temporal selectivity, such as driving or reading. It also presents a challenge to models of temporal selectivity that view such differences as statistical noise. Finally, the current investigation highlights the importance of a diachronic perspective (e.g., Reeves & Sperling, 1986; Wyble et al., 2011). Standard models of attention view attentional selection as a temporally discrete all-or-none process that neatly divides processing to a “pre-attentive” stage and an “attentive” stage (Neisser, 1967). The diachronic view eliminates this division (Zivony & Eimer, 2022a), as it describes selective attention as emerging from multiple processes that modulate visual perception gradually and continuously in real time. Such a perspective is critical to understanding temporal binding errors, which cannot be adequately accounted for with standard attention accounts. Further research into temporal binding can thus benefit cognitive research more generally as this line of inquiry can challenge long-held assumptions about the functional and temporal organisation of attentional mechanisms in vision.

## Footnotes

1 We thank Julie Golomb for suggesting this analysis.

2 In both experiments, all effects reported below were also reliable when identity APIs were entered as the dependent variable with colour report as the independent variable.

3 We note that both the trial-by-trial and between-participants correlations were substantially larger in Experiment 2 than the equivalent correlations in Experiment 1 (*r* = .29-30 vs. *r* = .13 and *r* = .88-89 vs. *r* = .58, respectively). Among the possible reasons for this discrepancy are the reduced set size for target colours and identities, the change in task instructions, the increase in the number of trials, the fact that pre-target distractors were always response-irrelevant, or the fact that this experiment was run in the lab rather than online. Any of these factors may have increased reliability of Experiment 2 and/or strengthened the dependency between co-occurring features relative to Experiment 1.
